# Period of Life at Which Tuberculous Infection Occurs

**Published:** 1916-05

**Authors:** 


**Period of Life at Which Tuberculous Infection Occurs.** S. Adolphus Knopf, Med. Rec., Jan. 8, from replies received from 60 authorities consulted gives the following statistics of the time at which tuberculosis was contracted: Under 1 year, 1-9%; 1-3 years, 9-50%; 3-5 years, 27-75%; 6-10 years, 75%; 11-15 years, 12-94%. These estimates (minima and maxima of authorities consulted) are of course mainly opinions though to some extent based on careful histories and serial statistics. All agree that the lungs and lymph nodes are the parts most frequently affected primarily and that an infection in infancy or early childhood most frequently becomes active at about the 15th year, next most frequently between 18 and 30. Excluding early infection, the disease appears and is apparently actually contracted in most cases in the period 20-35 years.

